# Nanoporous Ge thin film production combining Ge sputtering and dopant implantation

**DOI:** 10.3762/bjnano.6.32

**Published:** 2015-01-30

**Authors:** Jacques Perrin Toinin, Alain Portavoce, Khalid Hoummada, Michaël Texier, Maxime Bertoglio, Sandrine Bernardini, Marco Abbarchi, Lee Chow

**Affiliations:** 1 Aix-Marseille University, IM2NP, Faculté des Sciences de Saint-Jérôme case 142, 13397 Marseille, France; 2 CNRS, IM2NP, Faculté des Sciences de Saint-Jérôme case 142, 13397 Marseille, France; 3 Department of Physics, University of Central Florida, Orlando, FL 32816, USA

**Keywords:** germanium, ion implantation, porous material

## Abstract

In this work a novel process allowing for the production of nanoporous Ge thin films is presented. This process uses the combination of two techniques: Ge sputtering on SiO_2_ and dopant ion implantation. The process entails four successive steps: (i) Ge sputtering on SiO_2_, (ii) implantation preannealing, (iii) high-dose dopant implantation, and (iv) implantation postannealing. Scanning electron microscopy and transmission electron microscopy were used to characterize the morphology of the Ge film at different process steps under different postannealing conditions. For the same postannealing conditions, the Ge film topology was shown to be similar for different implantation doses and different dopants. However, the film topology can be controlled by adjusting the postannealing conditions.

## Introduction

Porous materials are of great interest for a large scope of industrial applications dealing with adsorption, catalysis, or molecular filtration and isolation. Furthermore, porous semiconductors can exhibit interesting properties for optoelectronic applications. For example, porous Si was shown to exhibit an increased band gap compared to bulk Si due to quantum (Q) size effects, related either to the formation of pseudo Q-wires or Q-dots in the porous structure, depending on the production method [1].

Generally, porous Si photoluminescence data can be interpreted either with a Q-wire model, or a model between the Q-wire and Q-dot models [2]. Porous Si optoelectronic properties were shown to be mainly determined by the “skeleton size” of the material and not by the pore sizes. However, in some cases, controlling the pore size allows for the control of the skeleton size, and thus should allow semiconductor band gap engineering, where the aim is the design of devices able to absorb or emit light at a tunable wavelength. Efficient visible electroluminescence has been achieved with porous Si for different wavelengths (red–green). Si and Ge are indirect gap materials, requiring phonon scattering for optical absorption/emission to take place. However, Q-size effects present in porous semiconductors can promote optical transitions without the need of phonons by breaking the momentum conservation rules and/or by making the material quasi-direct through the process of Brillouin zone folding [3]. For example, non-phonon processes were shown to dominate in the case of porous Si under strong confinement potential [4–6]. In addition, Q-effects in porous semiconductors can be interesting for photovoltaic applications, since they can lead to multiple exciton generation [7]. In particular, multiple exciton generation has been previously demonstrated in Si nanostructures [8].

Ge has a similar structure to Si, however, it offers several benefits compared to Si such as faster carrier mobility, smaller band gap and lower process temperatures [9]. In addition, the Ge exciton Bohr radius (≈24 nm) is significantly larger than that of Si (≈4.5 nm), allowing for quantum effects to appear in nanostructures exhibiting larger sizes [10], and allowing the k-selection rules to be broken. For example, lasing has only been observed in Ge for the case of strained [11] and doped [12] Ge layers. Furthermore, in addition to its small indirect band gap (≈0.66 eV), Ge exhibits a larger direct band gap (≈0.80 eV) that could promote non-phonon optical transitions if n-type doping of about 10^20^ cm^−3^ could be achieved in Ge. Since Ge is compatible with complementary metal oxide semiconductor (CMOS) technology, the production of porous Ge thin films could be used for integration of optoelectronic devices in Si microelectronic technology.

The production of porous Ge can be performed using several techniques such as anodization and electrochemical etching, spark processing or inductively coupled plasma chemical vapor deposition [13–15].

Ion implantation is a well-known technique used in the microelectronic industry to dope the active regions of semiconductor devices. Generally, implantation leads to the formation of defects of different nature in the material (vacancy, dislocation, amorphization, etc.). High-dose implantations in Ge (>10^15^ atoms/cm^2^) have been reported to induce the formation of nanoporous structures [16–25]. Thus, ion implantation may be a simple way to produce a nanoporous semiconductor.

In the present work, the impact of high dose selenium and tellurium (3.5 × 10^15^ atoms/cm^2^) implantations on the morphology of polycrystalline Ge thin films is presented, as well as the evolution of the film morphology with thermal annealing conditions (temperature and time).

## Results and Discussion

**Figure 1:**
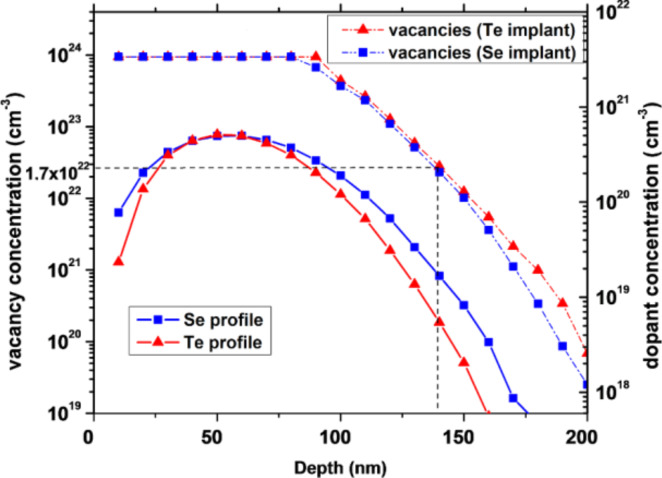
SRIM calculations of the implant distribution of Te (red) and Se (blue) atoms in Ge. The distributions of implanted ions are shown using straight lines on the right axis and the vacancies distributions are shown using dashed lines on the left axis.

340 nm thick Ge layers were deposited on the native oxide layer of a silicon substrate at room temperature (RT), under high vacuum, by magnetron sputtering. Recrystallization was then performed by rapid thermal annealing at 600 °C under vacuum (*P* ≈ 3 × 10^−5^ mbar) and the Ge layer was implanted under vacuum (*P* ≈ 2 × 10^−6^ mbar). Three types of implantations were performed: (i) the first set of samples were implanted with a 3.6 × 10^15^ atoms/cm^2^ dose of Se atoms with an energy of 130 keV, (ii) the second set of samples were implanted with a 3.1 × 10^15^ atoms/cm^2^ dose of Te atoms with an energy of 180 keV, and (iii) the last set of samples were co-implanted with both Se and Te atoms under the same conditions as previously mentioned. Figure 1 shows the predicted dopant and vacancy concentration profiles induced by implantation using the Stopping and Range of Ions in Matter (SRIM) software. This software is used in the ion implantation research and technology community to predict implantation profiles as well as implantation-induced defect distributions given the implantation energy, the nature of the implanted species, and the nature of the substrate [26]. The calculations are based on the classical theories of the stopping of ions in matter and Monte Carlo simulations: the energy loss of ions in matter are calculated and used to provide stopping powers, range and straggling distributions of implanted ions. In addition, the kinetic effects associated with the physics of implantation-mediated defects are also taken into account, allowing the distribution of point defects created in the target material to be obtained [27–28].

The dopant distributions follow a Gaussian distribution with a maximum concentration of 5 × 10^20^ atoms/cm^3^ located at a depth of 55 nm. Given that the more Ge-rich, Ge–Se compound is GeSe [29] and the only Ge–Te compound is GeTe [30], the presently studied implantations (exhibiting a maximum concentration level of about 1%) are not expected to allow the formation of a full compound layer. However, the formation of small Ge-dopant clusters is possible (the Se–Te binary system is fully miscible, corresponding to an ideal solution [31]). Usually, a damage energy higher than 5 eV/atoms^1^ corresponding to a vacancy concentration of ≈1.7 × 10^22^ vac/cm^3^ leads to Ge amorphization [32]. Thus, the SRIM calculations predict the formation of an amorphous Ge layer from the surface of the Ge film up to a depth of 140 nm. One can observe in Figure 1 that no dopants and no vacancies are expected to be found at a depth larger than 220 nm. Consequently, the implantation-induced defects should be confined in the Ge layer thickness.

**Figure 2:**
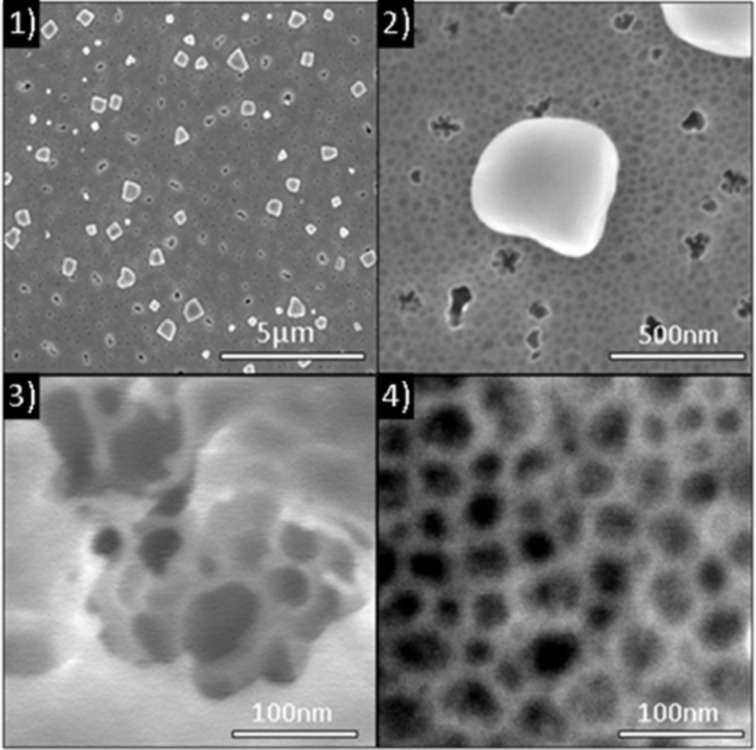
SEM plan-view images of the as-implanted Se sample: (1) low resolution view showing the different types of defects; (2) a single GeO*_x_* cluster; (3) the structure of holes; and (4) the nanoporous structure.

Figure 2 presents scanning electron microscopy (SEM) plan-view images of the as-implanted Se sample. The implantation induces the formation of three types of defects, randomly distributed on or in the germanium layer: (i) large clusters of Ge oxide with an average lateral size of ≈400 nm (composition analyzed by atom probe tomography, not reported here), (ii) holes with an average lateral size of ≈100 nm, and (iii) a nanoporous structure exhibiting pores with an average lateral size of ≈35 nm. The same three types of defects are observed in all the three sets of samples.

The average size and the density of these defects are reported in Table 1. An increase of the size of holes and of the clusters can be noticed between the Se-only implanted sample and the other sets of samples (60% for holes and 70% for clusters). However, the impact of the co-implantation is limited compared to the individual implantations. Furthermore, it can be noted that the porous structure is independent of the implantation type. Thus, variation in the defects appears to be dependent on the mass and the energy of the implanted species, but independent of the implanted dose.

**Table 1:** Surface density and average lateral size of the different implantation-induced defects versus implanted species.

Defect type	Property	Cluster	Hole	Porous structure

Se implantation	Average size (nm)	360 ± 2	70 ± 4	32 ± 2
	Density (×10^6^ cm^–2^)	4.2 × 10^1^ ± 3.5	1 × 10^3^ ± 70	5.49 × 10^4^ ± 2.5 × 10^2^
Te implantation	Average size (nm)	520 ± 8	120 ± 1	33 ± 2
	Density (×10^6^ cm^–2^)	3 ± 1	5.7 × 10^2^ ± 90	4.35 × 10^4^ ± 2.5 × 10^2^
Se and Te co-implantation	Average size (nm)	540 ± 3	130 ± 3	40 ± 1
	Density (×10^6^ cm^–2^)	3.7 × 10^1^ ± 4	5.69 × 10^2^ ± 90	3.70 × 10^4^ ± 1 × 10^2^

Various thermal postannealing treatments were performed after ion implantation. For a better understanding of the evolution of the film morphology with the thermal treatments, the different annealing treatments are compared using a reference scale defined as the thermal budget (TB) based on the surface diffusion length of Ge atoms on the silicon (111) surface described by:

[1]
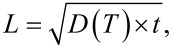


where *L* is the Ge surface diffusion length (cm), and *D* = 0.06 × exp(−2.47 eV/*kT*) is the Ge surface diffusion coefficient (cm^2^ s^−1^) on Si [33], depending on both the temperature and the annealing time, *t*. Table 2 presents the annealing parameters. One can distinguish three ranges of thermal treatments: (i) a low annealing TB with TB = 3.1 µm, (ii) an intermediate TB between 3.9 µm and 4.8 µm, and (iii) a high TB = 8.7 µm.

**Table 2:** Thermal annealing conditions (temperature and time), and corresponding thermal budgets.

Thermal budget	Low → High				

Temperature (°C)	525	625	675	575	725
Time (h)	168	5	1	48	1
Diffusion length (µm)	3.1	3.9	4.1	4.8	8.7

**Figure 3:**
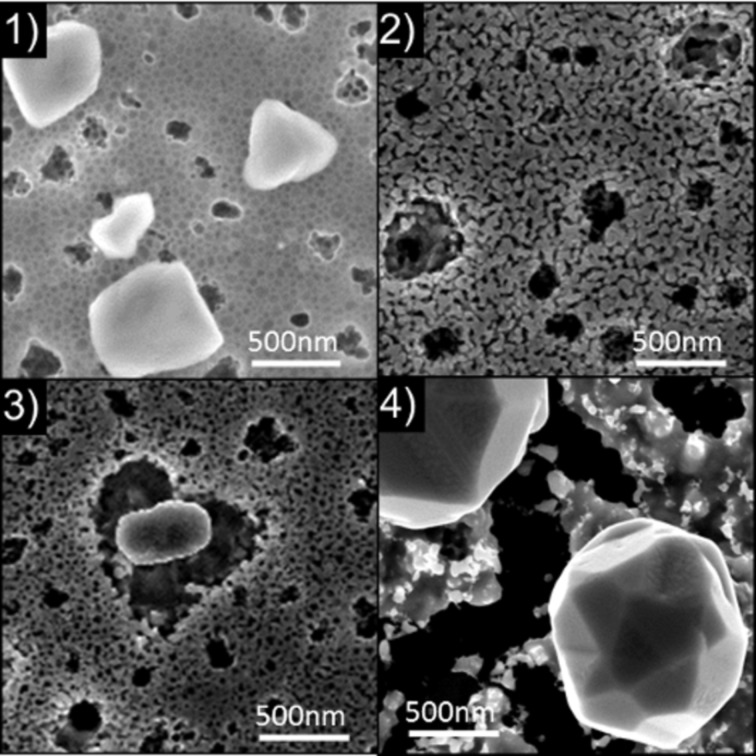
Thermal annealing effects on the co-implanted Se/Te sample: (1) as-implanted, (2) TB = 4.1 µm; (3) TB = 4.8 µm; and (4) TB = 8.7 µm.

Figure 3 presents the influence of the thermal annealing on the morphology of the co-implanted Se/Te sample. Thermal treatments induce large modifications of the implantation-induced defect morphology. For 4.1 ≤ TB ≤ 4.8 µm (Figure 3.2 and 3.3), the GeO*_x_* clusters initially observed on the as-implanted film surface (Figure 3.1) vanished during annealing. Instead, large holes can be observed (Figure 3.2), with a depth as deep as the Ge film thickness. These holes exhibit the same size and the same surface density as the initial clusters, leading to the conclusion that they are actually located on the surface sites initially occupied by a GeO*_x_* cluster. The initial holes present in the as-implanted film still exist after annealing. However, the initial nanoporous structure experienced a morphology modification leading to an increase of the pore size. In addition, new types of clusters appeared on the surface (Figure 3.3). They are characterized by a surrounding trench that is typical of crystal growth which uses the surrounding material and is limited by atomic surface diffusion. At this thermal budget, a three-scale porous structure is obtained with (i) large holes linked to the disappearance of the GeO*_x_* clusters (average lateral size ≈500 nm), (ii) the holes initially present in the as-implanted samples (average lateral size ≈100 nm), and (iii) the modified nanoporous structure (average lateral size ≈50 nm). For the highest thermal budget (TB = 8.7 μm, Figure 3.4), the structure of the Ge film is greatly modified. One can observe the disappearance of both the holes and the nanoporous structure. Instead, the SEM plan-view analysis (Figure 3.4) reveals the growth of faceted crystallites, with an average lateral size of 700 nm for the Se-implanted sample and of 1600 nm for the co-implanted sample, and a complex surface structure between these crystallites. Indeed, some parts of the surface exhibit large roughness, while some others appear completely flat (black contrast in Figure 3.4). This phenomenon can be explained considering that these crystallites result from the Ge dewetting mechanism occurring on the buried SiO_2_ layer already observed in Figure 3.3. The general dewetting phenomenon is due to surface/interface energy minimization between the film and the substrate, leading to island formation or agglomeration at a temperature below the melting temperature of the film material. This phenomenon is generally undesirable in the field of micro- or nano-technology [34] yet has been reported to be interesting for the fabrication of nanocrystals. A wide range of materials can be used for the fabrication of nanocrystals by dewetting, such as metals or semiconductors. In addition, the structure of the dewetted layers can be controlled using several techniques such as pulsed laser annealing [35–36] or a substrate patterned by focused ion beam. The study of Ge dewetting on SiO_2_ [37] has already been reported in the literature, however, only in the case of very thin amorphous Ge layers (5−15 nm thick) [38–40]. For a large TB, the atomic diffusion length on the surface is significant, and during dewetting, Ge atoms can form large crystallites. In this case, between the crystallites, the very flat parts of the surface correspond to the flat SiO_2_ layer that is revealed due to the dewetting phenomenon, while the rough parts of the surface correspond to the surface regions where the dewetting phenomenon is incomplete.

**Figure 4:**
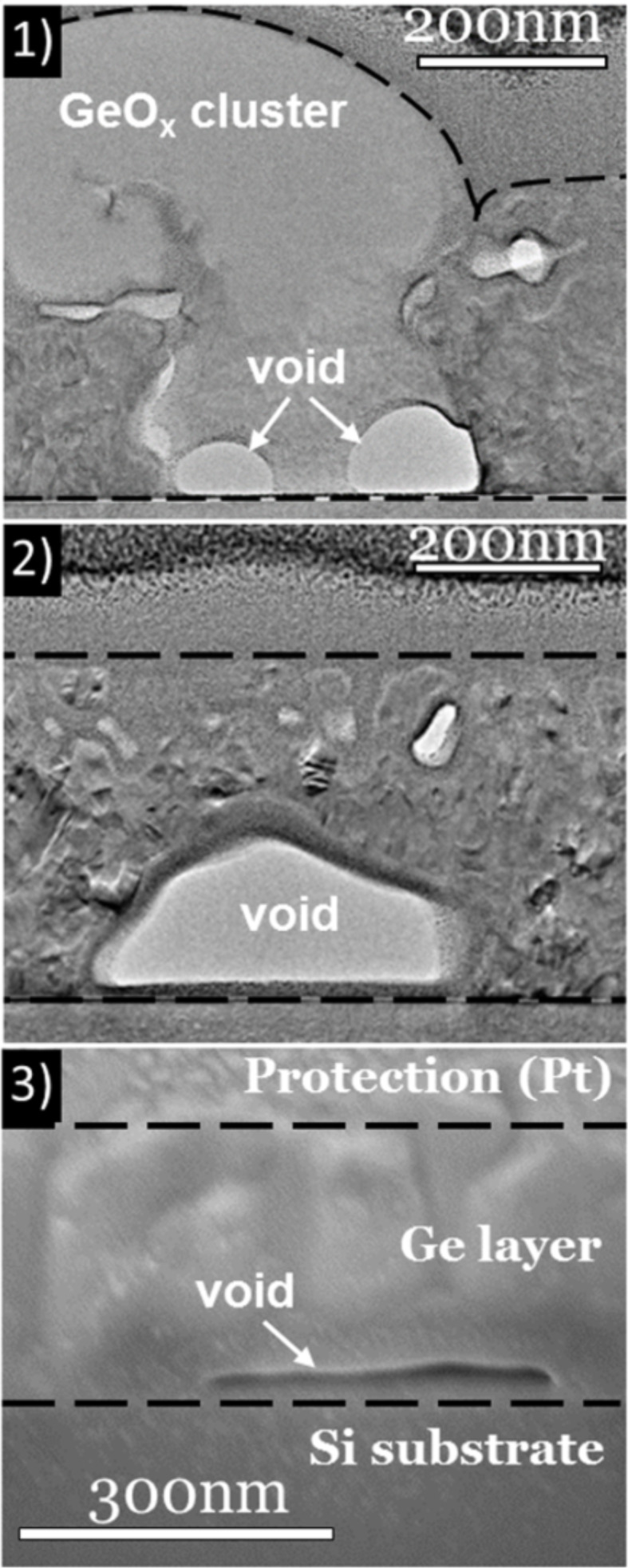
(1) and (2) TEM cross-sectional view of the Se-implanted sample after annealing with TB = 3.1 µm. (3) SEM cross-sectional view of the Se-implanted sample after annealing with TB = 4.8 µm.

The cross-sectional analysis shown in Figure 4 gives interesting information about the observed implantation-induced defects, and confirms the evolution of the nanoporous structure and the holes between the lowest thermal budget (TEM analysis, Figure 4.1 and 4.2) and TB = 4.8 µm (SEM analysis, Figure 4.3).

A detailed analysis of the images indicates that the brightest areas (identified as being GeO*_x_* clusters) correspond to amorphous materials that are in contact with the buried native SiO_2_ layer, present through the entire Ge layer thickness. Therefore, the disappearance of the GeO*_x_* clusters can be explained by the complete evaporation of the GeO*_x_* during annealing, leaving deep holes in the Ge film. The image contrast is affected by the variation of both local diffraction conditions and absorption. The moiré pattern visible in various areas of the deposited layer confirms its polycrystalline structure. The pores are difficult to observe in the cross-sectional view due to the superimposition of the structure in the analysis and due to the filling of the pores by the protective Pt layer, however, the various Ge nanograins (≤50 nm) exhibiting different orientations are easily observed. The SEM cross-sectional view of the sample with a TB = 4.8 µm (Figure 4.3) shows the porosity enlargement of the porous structure compared to lower TB (Figure 4.2). In addition to the implantation-induced defects identified in plan-view observations, cross-sectional observations show the existence of cavities at the Ge/SiO_2_ interface (Figure 4.1 and 4.2). These cavities present facets when in contact with polycrystalline Ge, and present a spherical shape when in contact with amorphous GeO*_x_*. They can be related to the initial Ge dewetting mechanism, and thus, are expected to form during annealing.

Figure 5.1 presents an SEM plan-view image obtained on a 340 nm thick Ge film without implantation, but annealed in an RTP furnace at *T* ≈ 650 °C for 20 min. One can note that even without implantation, the Ge film is dewetted on the SiO_2_ layer, exhibiting a net shape. Consequently, the dewetting phenomenon should play a significant role in the atomic redistribution observed during annealing of implanted films. However, the structure obtained with the as-deposited Ge films (Figure 5.1) is quite different from the structure obtained with implanted films (Figure 3 and Figure 5.2). Consequently, the implantation process, and possibly the nature of the implanted dopants (Ge-dopant cluster formation, surface and interface segregation, etc.) as well as their atomic diffusion mechanism in the bulk and on the surface of the Ge film, have a significant effect on the Ge dewetting phenomenon. For example, the cavity formation at the Ge/SiO_2_ interface could be also related to the diffusion mechanism of Se and Te atoms [41].

**Figure 5:**
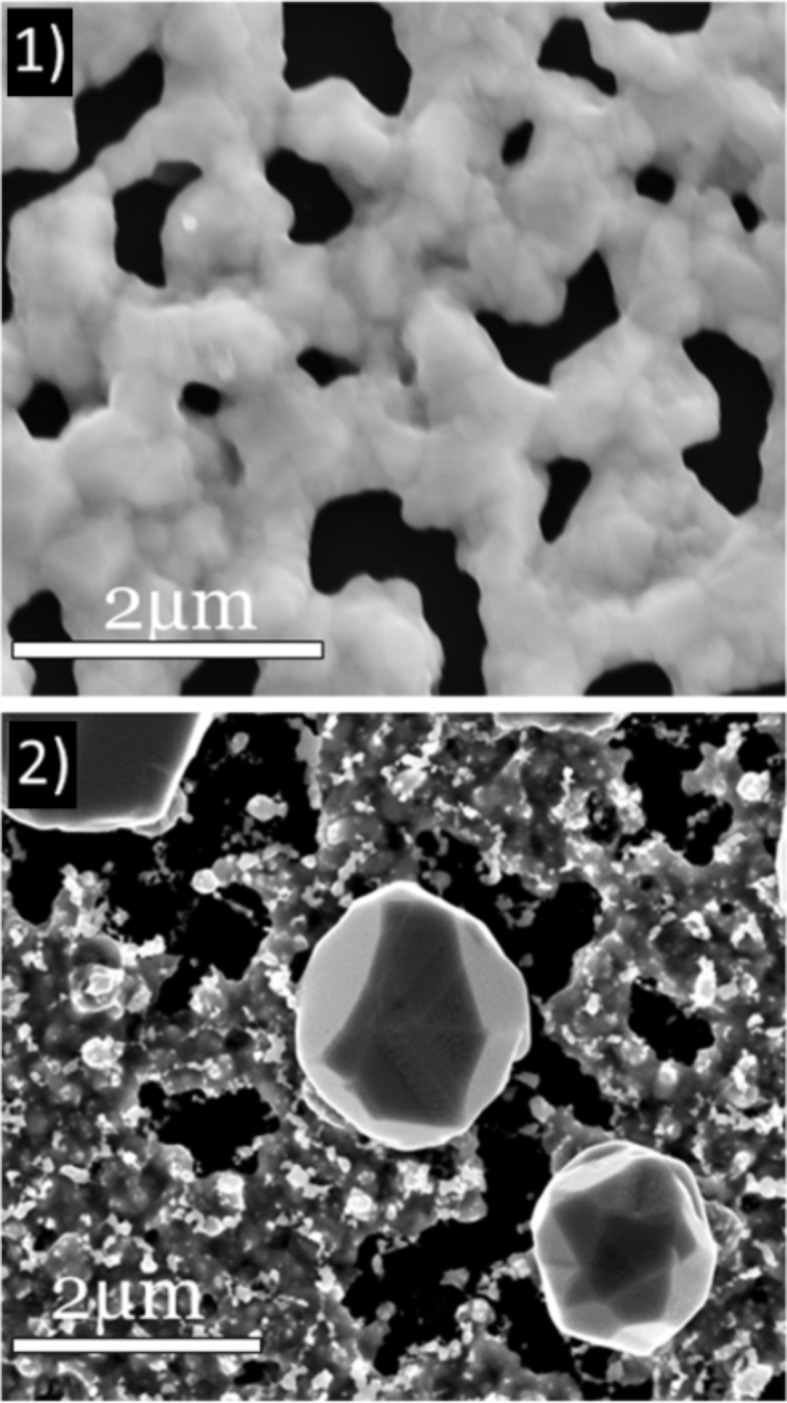
SEM plan-view image obtained after annealing a 340 nm thick Ge layer sputtered on the native Si oxide of a Si(001) substrate: (1) without implantation and TB = 0.64 µm; and (2) with Se implantation and TB = 8.7 µm.

## Conclusion

The fabrication of highly-doped, porous Ge thin films (which are of high potential use for optoelectronic device fabrication) was successfully achieved using experimental techniques compatible with Si CMOS technology.

High-dose (>10^15^ atoms/cm^2^) dopant implantations (Se and Te) have been performed in polycrystalline Ge films deposited on the native Si oxide. These implantations induced the formation of three types of defects in the Ge film: (i) large GeO*_x_* clusters, (ii) holes, and (iii) nanopores (≈35 nm wide). Under thermal annealing (i) the large GeO*_x_* clusters disappear leaving large pores (≈400 nm wide) in the film, (ii) the initial holes stay quasi-unchanged in the film, forming ≈100 nm wide pores, and (iii) the size of the nanopores increases (≈50 nm wide). In addition, cavities can form at the Ge/SiO_2_ interface with a lateral size of between 100 and 200 nm. At high thermal budget, the film is completely fragmented with the formation of large Ge islands (≈1 µm wide). These phenomena can be explained by the combination of several mechanisms. Among them, the Ge dewetting on the SiO_2_-buried layer is the most obvious. However, the influence of the nature of the implanted dopants (bulk and surface diffusion) and the possible formation of Ge-dopant nanoclusters can also have a significant effect on the observed atomic redistribution. The careful control of thermal annealing conditions should allow the control of the size and of the distribution of the pores, allowing for the production of Ge nanoporous films exhibiting characteristic skeleton sizes smaller (≈10 nm, without annealing) or larger (≈50 nm, annealing at 675 °C for 1 h) than the Ge-exciton Bohr radius, depending on the desired applications.

## Experimental

The Ge layers were deposited on the native silicon oxide of a (001) silicon wafer by magnetron sputtering in a commercial set up with a deposition chamber exhibiting a base pressure of ≈10^−8^ mbar. The first thermal annealing executed after Ge deposition was performed in a commercial Jetfirst 600 Rapid Thermal Annealing furnace under a vacuum of 2 × 10^−5^ mbar at *T* = 600 °C for 20 min. The samples were implanted with a dose of 3.6 × 10^15^ atoms/cm^2^ using the industrial implanter IMC200 developed by the company IBS. The implantations were performed under a pressure of 2 × 10^−6^ mbar at an angle of 7° with respect to the normal of the sample surface, and with an ion beam energy of 130 keV for Se^+^ ions and of 180 keV for Te^+^ ions. After implantation, the samples were annealed in different conditions (1 ≤ *t* ≤ 168 h and 525 ≤ *T* ≤ 725 °C) in a custom-built furnace under a pressure of 1 × 10^−7^ mbar during annealing.

TEM images were performed using a FEI Titan 80-300 Cs-corrected microscope operating at 200 kV under multibeam conditions with the Ge substrate aligned along the <110> crystallographic direction. The spherical aberration was tuned to −15 µm to both optimize the spatial resolution and reduce the spatial delocalization [42].

SEM images were performed using a FEI Helios 600 Nanolab microscope in the secondary electron (SE) mode with an accelerating voltage of 5 kV, using either an Everhart–Thornley Detector (ETD) located below the objective lens for imaging at low magnifications, or using a through lens detector (TLD) placed within the objective lens for capturing high-resolution images. In this mode, the image contrast is mainly affected by topographic variations allowing the presence of holes and asperities at the sample surface to be evidenced. The lateral size and the density were analyzed manually from the SEM images using the ImageJ software developed at the National Institute of Health. The errors in the measurements can be estimated with the original SEM image resolution.

## References

[1] Schuppler S, Friedman S L, Marcus M A, Adler D L, Xie Y-H, Ross F M, Chabal Y J, Harris T D, Brus L E, Brown W L (1995). Phys Rev B.

[2] Barbagiovanni E G, Lockwood D J, Simpson P J, Goncharova L V (2014). Appl Phys Rev.

[3] Iyer S S, Xie Y-H (1993). Science.

[4] Hybertsen M S (1994). Phys Rev Lett.

[5] Kovalev D, Heckler H, Ben-Chorin M, Polisski G, Schwartzkopff M, Koch F (1998). Phys Rev Lett.

[6] Kovalev D, Heckler H, Polisski G, Diener J, Koch F (2001). Opt Mater.

[7] Nozik A J (2012). Nat Photonics.

[8] Beard M C, Knutsen K P, Yu P, Luther J M, Song Q, Metzger W K, Ellingson R J, Nozik A J (2007). Nano Lett.

[9] Sze S M (1981). Physics of Semiconductor Devices.

[10] Barbagiovanni E G, Lockwood D J, Simpson P J, Goncharova L V (2012). J Appl Phys.

[11] Süess M J, Geiger R, Minamisawa R A, Schiefler G, Frigerio J, Chrastina D, Isella G, Spolenak R, Faist J, Sigg H (2013). Nat Photonics.

[12] Liu J, Sun X, Camacho-Aguilera R, Kimerling L C, Michel J (2010). Opt Lett.

[13] Choi H C, Buriak J M (2000). Chem Commun.

[14] Chang S-S, Hummel R E (2000). J Lumin.

[15] Ko T S, Shieh J, Yang M C, Lu T C, Kuo H C, Wang S C (2008). Thin Solid Films.

[16] Wilson I H (1982). J Appl Phys.

[17] Wang L M, Birtcher R C (1989). Appl Phys Lett.

[18] Stritzker B, Elliman R G, Zou J Nucl Instrum Methods Phys Res, Sect B.

[19] Wang L M, Birtcher R C (1991). Philos Mag A.

[20] Holland O W, Appleton B R, Narayan J (1983). J Appl Phys.

[21] Ottaviano L, Verna A, Grossi V, Parisse P, Piperno S, Passacantando M, Impellizzeri G, Priolo F (2007). Surf Sci.

[22] Romano L, Impellizzeri G, Tomasello M V, Giannazzo F, Spinella C, Grimaldi M G (2010). J Appl Phys.

[23] Janssens T, Huyghebaert C, Vanhaeren D, Winderickx G, Satta A, Meuris M, Vandervorst W (2006). J Vac Sci Technol, B.

[24] Romano L, Impellizzeri G, Bosco L, Ruffino F, Miritello M, Grimaldi M G (2012). J Appl Phys.

[25] Darby B L, Yates B R, Rudawski N G, Jones K S, Kontos A, Elliman R G (2011). Thin Solid Films.

[26] (2014). Particle interactions with matter.

[27] Ziegler J F, Biersack J P, Littmark U (1985). The Stopping and Range of Ions in Solids.

[28] Ziegler J F (2004). Nucl Instrum Methods Phys Res, Sect B.

[29] Ipser H, Gambino M, Schuster W (1982). Monatsh Chem.

[30] Okamoto H (2000). J Phase Equilib.

[31] Ghosh G, Sharma R C, Li D T, Chang Y A (1994). J Phase Equilib.

[32] Claverie A, Koffel S, Cherkashin N, Benassayag G, Scheiblin P (2010). Thin Solid Films.

[33] Allen C E, Ditchfield R, Seebauer E G (1997). Phys Rev B.

[34] Thompson C V (2012). Annu Rev Mater Res.

[35] Trice J, Thomas D, Favazza C, Sureshkumar R, Kalyanaraman R (2007). Phys Rev B.

[36] Ruffino F, Pugliara A, Carria E, Romano L, Bongiorno C, Spinella C, Grimaldi M G (2012). Nanotechnology.

[37] Abbarchi M, Naffouti M, Vial B, Benkouider A, Lermusiaux L, Favre L, Ronda A, Bidault S, Berbezier I, Bonod N (2014). ACS Nano.

[38] Szkutnik P D, Karmous A, Bassani F, Ronda A, Berbezier I, Gacem K, El Hdiy A, Troyon M (2008). Eur Phys J: Appl Phys.

[39] Gacem K, El Hdiy A, Troyon M, Berbezier I, Ronda A (2010). Nanotechnology.

[40] Berbezier I, Aouassa M, Ronda A, Favre L, Bollani M, Sordan R, Delobbe A, Sudraud P (2013). J Appl Phys.

[41] De Luca A, Portavoce A, Texier M, Grosjean C, Burle N, Oison V, Pichaud B (2014). J Appl Phys.

[42] Texier M, Thibault-Pénisson J (2012). Micron.

